# Supplementary material for: Systemic and Airway Epigenetic Disruptions Are Associated with Health Status in COPD
Source: Biomedicines. 2023 Jan 5;11(1):134. doi: 10.3390/biomedicines11010134 (PMC9855774; doi:10.3390/biomedicines11010134)
Supplement: Supplementary file 1 [file biomedicines-11-00134-s001.zip › Figure S1-S3.pdf]

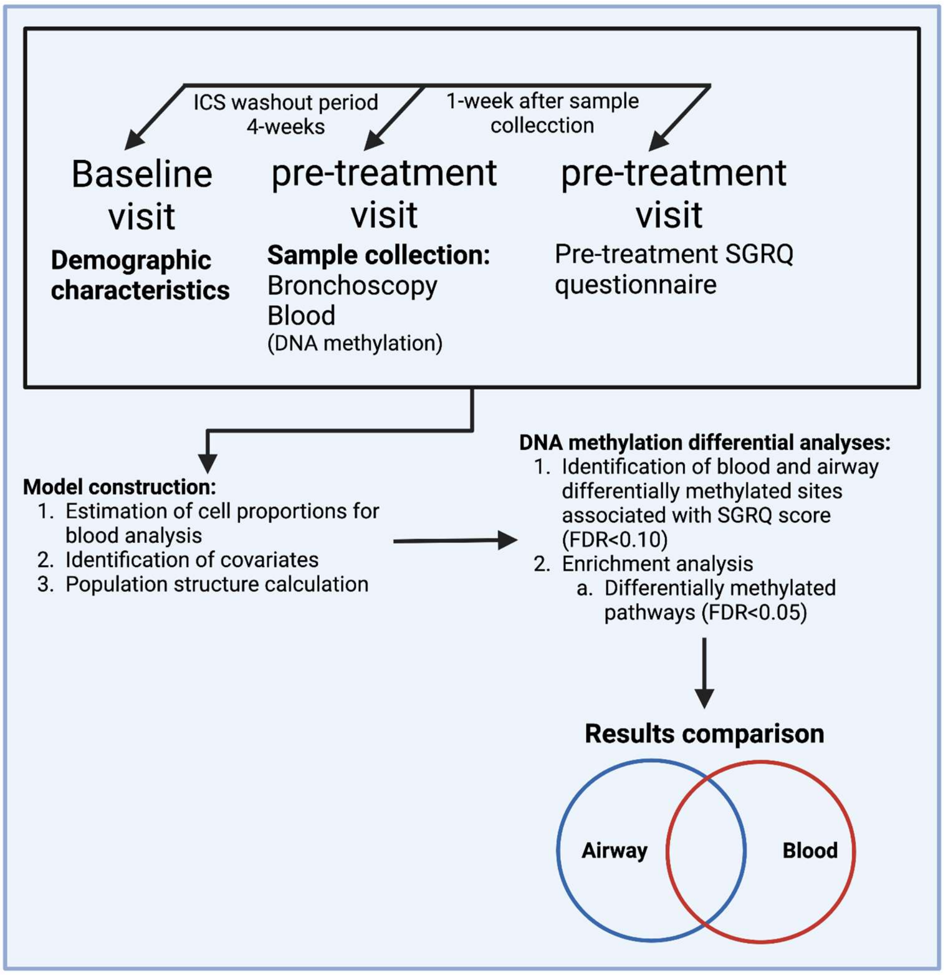

Supplemental Figure S1. Study design. SGRQ: St. George Respiratory Questionnaire

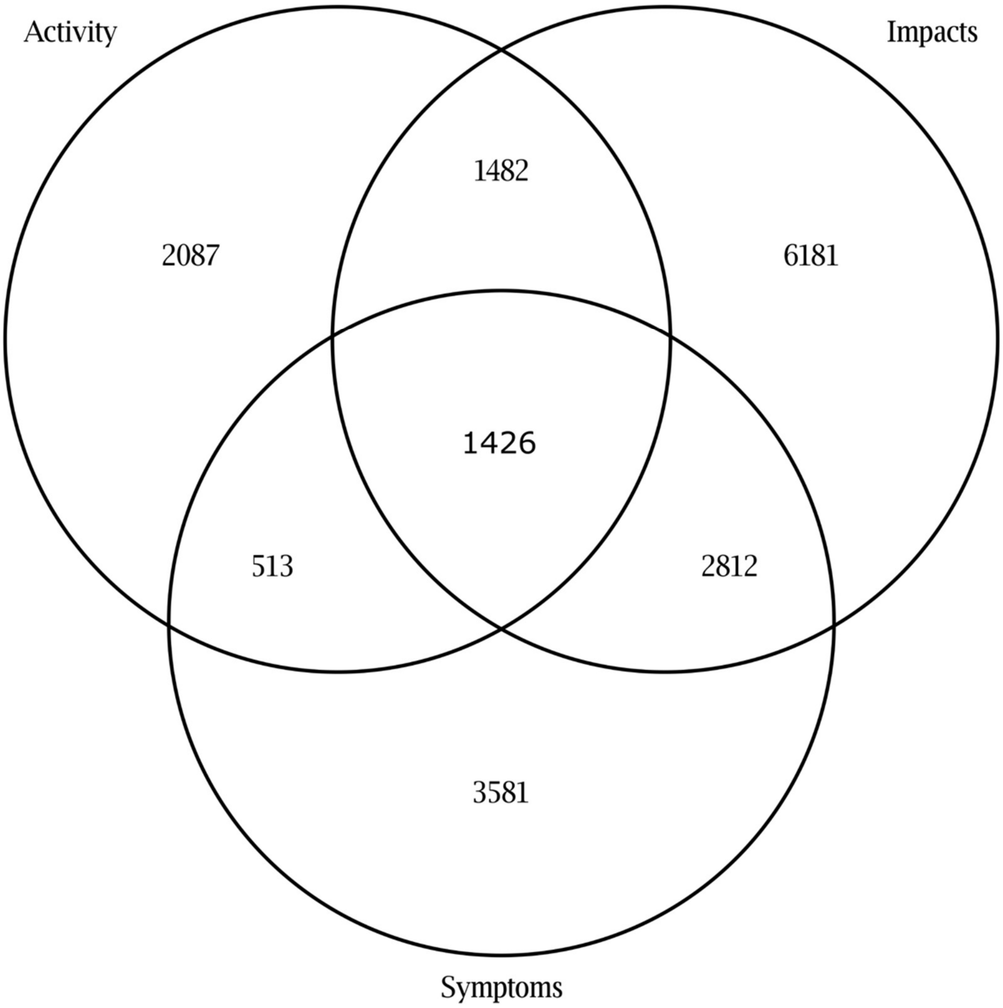

Supplemental Figure S2. Differentially methylated genes overlap between the three SGRQ domains. Blood analyses.

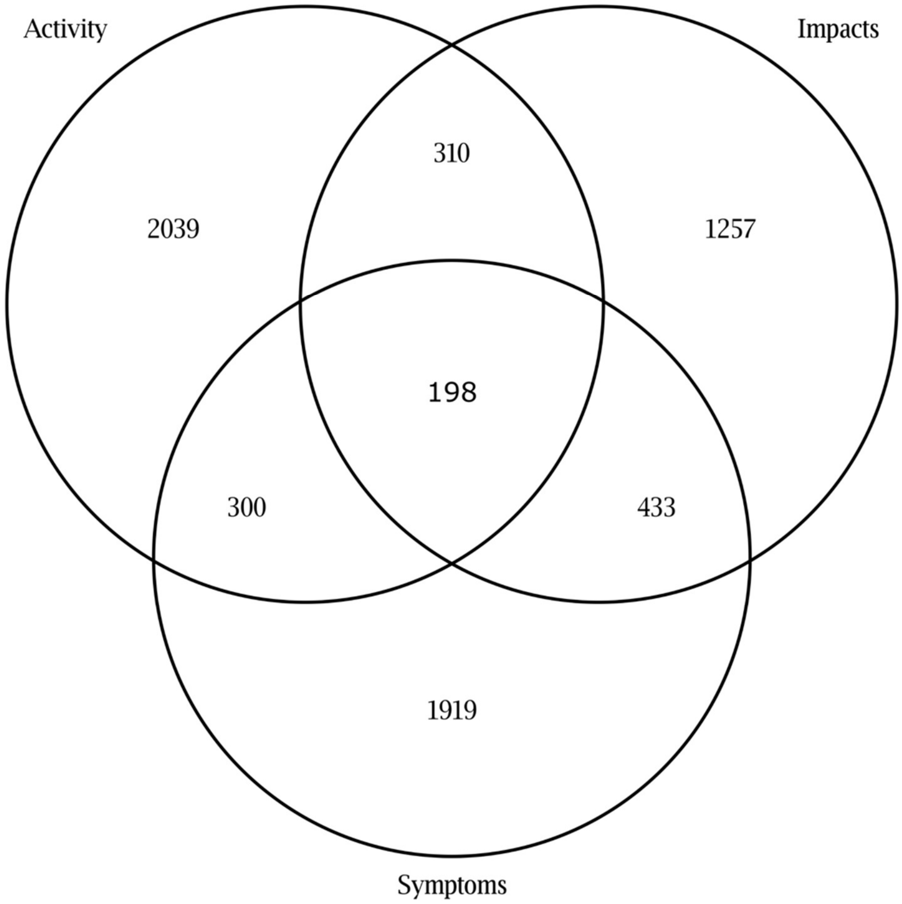

Supplemental Figure S3. Differentially methylated genes overlap between the three SGRQ domains. Airway analyses.
